# A common inducer molecule enhances sugar utilization by *Shewanella oneidensis* MR-1

**DOI:** 10.1093/jimb/kuad018

**Published:** 2023-08-03

**Authors:** Megan C Gruenberg, Michaela A TerAvest

**Affiliations:** Department of Biochemistry and Molecular Biology, Michigan State University, East Lansing, MI, 48824, USA; Department of Biochemistry and Molecular Biology, Michigan State University, East Lansing, MI, 48824, USA

**Keywords:** *Shewanella oneidensis*, N-acetylglucosamine, Metabolism, Synthetic biology

## Abstract

*Shewanella oneidensis* MR-1 is an electroactive bacterium that is a promising host for bioelectrochemical technologies, which makes it a common target for genetic engineering, including gene deletions and expression of heterologous pathways. Expression of heterologous genes and gene knockdown via CRISPRi in *S. oneidensis* are both frequently induced by β-D-1-thiogalactopyranoside (IPTG), a commonly used inducer molecule across many model organisms. Here, we report and characterize an unexpected phenotype; IPTG enhances the growth of wild-type *S. oneidensis* MR-1 on the sugar substrate N-acetylglucosamine (NAG). IPTG improves the carrying capacity of *S. oneidensis* growing on NAG while the growth rate remains similar to cultures without the inducer. Extracellular acetate accumulates faster and to a higher concentration in cultures without IPTG than those with it. IPTG appears to improve acetate metabolism, which combats the negative effect that acetate accumulation has on the growth of *S. oneidensis* with NAG. We recommend using extensive experimental controls and careful data interpretation when using both NAG and IPTG in *S. oneidensis* cultures.

## Graphical Abstract

**Graphical Abstract:**
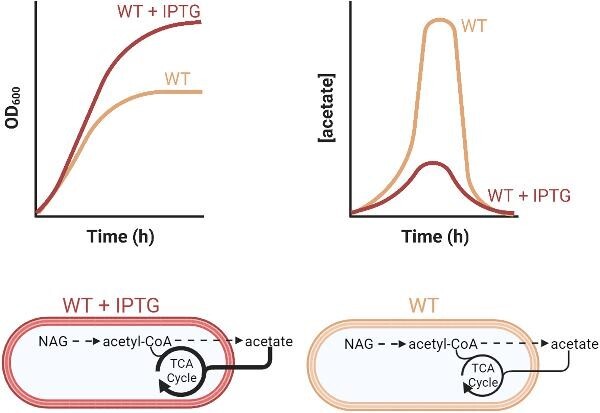
A chemical commonly used to induce expression of engineered genes enhanced sugar metabolism in a commonly studied bacterium.

## Introduction

*Shewanella oneidensis* MR-1 is a facultatively anaerobic bacterium with great potential as a host for bioelectrochemical technologies due to its well characterized extracellular electron transfer pathway and diverse genetic toolbox (Kouzuma, 2021; Zou et al., 2019). Some of these technologies include using *S. oneidensis* as a biosensor for arsenic or toxic water (Webster et al., 2014; Zang et al., 2021) and for electricity generation from waste (Lin et al., 2017; Min et al., 2017). However, *S. oneidensis* natively has limited options for carbon substrates and does not naturally accumulate high-value compounds (Venkateswaran et al., 1999). These limitations make pathway engineering a crucial component of this organism's expanded utility in bioelectrochemical systems.

Sugar metabolism is particularly limited in *S. oneidensis*, with several essential genes for sugar utilization missing or nonfunctional. For sugar transport, the *S. oneidensis* MR-1 genome encodes one set of phosphotransferase system genes for an unknown substrate, but phosphoenolpyruvate is not sufficiently produced to sustain the energy requirements for the system to function (Rodionov et al., 2010; Serres & Riley, 2006). Additionally, the genome does not encode an annotated glucose permease, glucose kinase, or the important glycolysis enzyme 6-phosphofructokinase, which prevents *S. oneidensis* from utilizing most six carbon sugars (Chubiz & Marx, 2017; Rodionov et al., 2010). N-acetylglucosamine (NAG) is a rare exception to this rule, as *S. oneidensis* MR-1 converts NAG to fructose 6-phosphate with a NAG permease, NAG kinase, NAG 6-phosphate deacetylase, and glucosamine 6-phosphate deaminase (Yang et al., 2006). With a limited pool of compounds that can be utilized for carbon and energy, *S. oneidensis* and its engineered strains are typically grown using lactate, pyruvate, acetate, or NAG as substrates.

Another prominent feature of *S. oneidensis* metabolism is acetate accumulation. Under anoxic conditions, *S. oneidensis* MR-1 excretes high levels of acetate because of limited tricarboxylic acid (TCA) cycle activity (Brutinel & Gralnick, 2012). Under oxic conditions, acetate accumulation and subsequent uptake are also observed in *S. oneidensis* cultures growing on NAG or lactate (Duhl et al., 2018; Feng et al., 2012). Acetate is a common product of overflow metabolism, and *S. oneidensis* begins to use the excreted acetate as a secondary substrate via the glyoxylate shunt when the main substrate is depleted (Feng et al., 2012). The accumulation of acetate is usually accompanied with a drop in media pH which can affect the health of the cells (Orr et al., 2019). In *S. oneidensis*, acetate is produced from acetyl-CoA and vice versa by the combined, reversible activities of acetate kinase and phosphate acetyltransferase; additionally, acetyl-CoA can be produced from acetate *via* acetyl-coenzyme A synthetase (Flynn et al., 2012).

To improve *S. oneidensis* as a host for use in bioelectrochemical systems, expansion of substrate utilization and product scope are critical. Gene expression under the control of an inducible promoter is one of the most common methods for experimenting with both native and non-native pathways *in vivo*. One tool that uses β-D-1-thiogalactopyranoside (IPTG)-inducible expression for studying gene function and for pathway engineering in *S. oneidensis* is the Mobile CRISPRi knockdown system (Peters et al., 2019). Promoters inducible by IPTG are utilized for both protein synthesis *via* common *S. oneidensis*-compatible vectors and CRISPRi knockdown systems. For example, Ford *et al*. deleted a gene previously thought to be essential in *S. oneidensis* (*gpmA*) by uncovering optimal knockout conditions using an IPTG-inducible CRISPRi system (Ford et al., 2022). *Shewanella oneidensis* has been engineered to utilize substrates, such as xylose and glucose via IPTG-inducible systems (Cheng et al., 2020; Li et al., 2017). Improved extracellular electron transfer in *S. oneidensis* has been achieved through various IPTG-inducible methods including using two types of transcriptional logic gates to fine-tune expression of extracellular electron transfer genes (Dundas et al., 2020), expression of NAD^+^ biosynthesis genes for increased intracellular NAD(H) levels (Li et al., 2018), expression of a synthetic flavin pathway from *Bacillus subtilis* to increase secreted flavins (Yang et al., 2015), and expression of diguanylate cyclase from *Escherichia coli* for enhanced *S. oneidensis* biofilm formation (Liu et al., 2015). IPTG-inducible systems have also been used for production of 5-aminolevulinic acid (Yi & Ng, 2021) and *n*-butanol (Jeon et al., 2018) in *S. oneidensis*, increasing the organism's potential product scope.

The use of IPTG for induction of heterologous genes is widespread due to the efficiency and simplicity of the system. The synthetic inducer is used to regulate the lac repressor (LacI), which regulates the *lac* operon in *E. coli*. Natively, the *lac* operon is expressed when lactose is present to produce lactose metabolic genes β-galactosidase, lactose permease, and a transacetylase (Lewis, 2011; Wilson et al., 2007). Lactose is detected indirectly by its isomer allolactose, which can bind to LacI (Lewis, 2011). LacI binds to the lac operator when allolactose concentrations are low, which physically blocks transcription of the *lac* operon. Allolactose induces transcription of the *lac* operon by binding to LacI and releasing it from the DNA (Jobe & Bourgeois, 1972). IPTG can be used as a replacement for allolactose since it is not hydrolyzed by cells, making it a gratuitous inducer whose concentration does not change during the course of the experiment (Wilson et al., 2007). When used in the context of heterologous gene expression, IPTG induces expression of a synthetic operon which is regulated by LacI. IPTG is an indispensable tool for genetic engineering of many organisms, *S. oneidensis* included. In this work, we characterize the unexpected phenotype of wild-type *S. oneidensis* MR-1 in which growth on the sugar substrate NAG is enhanced by the presence of IPTG and advise circumspection when using NAG and IPTG together in evaluation of engineered *S. oneidensis* strains.

## Results

IPTG-enhanced growth of wild-type *S. oneidensis* was discovered during experimentation with CRISPRi knockdown strains. With this system, a guide RNA is engineered to target a gene of interest. The guide RNA forms a complex with a catalytically inactive Cas9, which binds to the target gene and blocks its transcription (Peters et al., 2019). We observed that when grown on NAG, inducing the non-targeting control plasmid (pJMP2846) of the CRISPRi system with IPTG improved growth. To determine if this was due to the induction of the non-targeting control, we cultured wild-type *S. oneidensis* harboring pJMP2846 on 20 mM NAG with and without 10 mM IPTG and compared it to wild-type *S. oneidensis* without the plasmid under the same conditions (Fig. S1). We found that the improvement to growth was not exclusive to the CRISPRi non-targeting strain and explored the phenotype further in wild-type *S. oneidensis* carrying no heterologous plasmid.

**Fig. 1.**
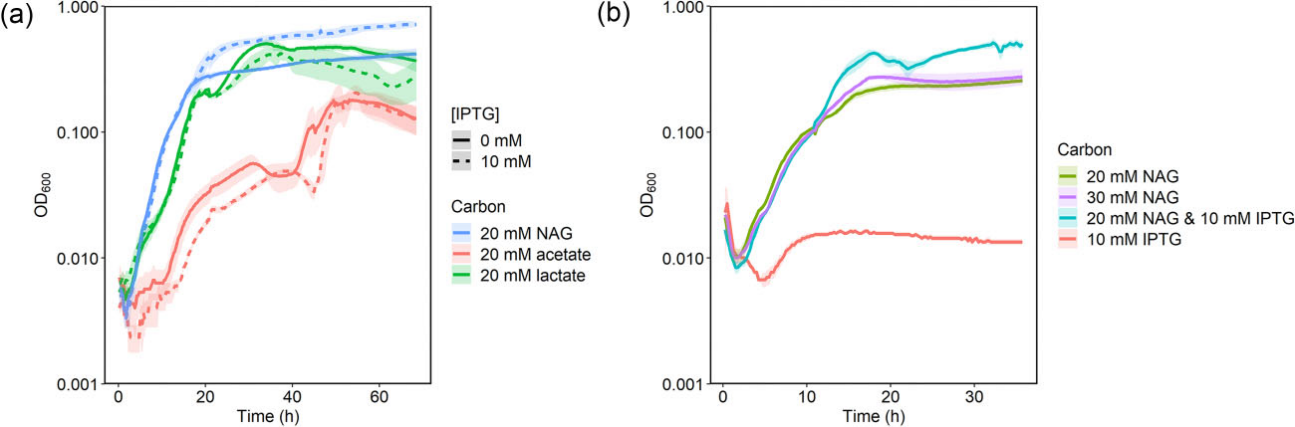
(a) Growth of *S. oneidensis* MR-1 cultivated on 20 mM of different carbon substrates with or without 10 mM IPTG in 200 µl M5 minimal medium in 96-well plates. (b) *Shewanella oneidensis* MR-1 cultivated on NAG and/or IPTG in 200 µl M5 minimal medium. *Y*-axis is on a logarithmic scale. Lines represent the average of three biological replicates and transparent ribbons represent standard error.

To determine if IPTG influenced *S. oneidensis* growth on various substrates, the wild-type strain was cultivated with 20 mM NAG, lactate, or acetate with and without 10 mM IPTG in 96-well plates (Fig. 1a). When *S. oneidensis* was grown on 20 mM NAG, the carrying capacity (final OD_600_) nearly doubled from 0.372 ± 0.002 without IPTG to 0.648 ± 0.004 with IPTG, while the growth rate remained similar at 0.181 ± 0.005 h^−1^ without IPTG and 0.168 ± 0.005 h^−1^ with IPTG (Fig. 1a, Table S1). IPTG slightly decreased the carrying capacity during growth on lactate but did not significantly affect the growth rate of MR-1 on either lactate or acetate (Table S1).

To determine whether the carrying capacity increased because *S. oneidensis* can consume IPTG as a carbon substrate, the wild-type strain was cultivated with 10 mM IPTG as the only available carbon source. *Shewanella oneidensis* was incapable of growing on IPTG alone. Additionally, *S. oneidensis* cultivated on 30 mM NAG did not share the same growth phenotype as when cultivated with 20 mM NAG and 10 mM IPTG (Fig. 1b), indicating that changes in osmotic pressure were not responsible for the increased carrying capacity. This observation also indicates that IPTG does not act as a secondary substrate, demonstrated by the lack of carrying capacity increase by 30 mM NAG as compared to the increase in carrying capacity of 20 mM NAG and 10 mM IPTG.

**Fig. 2.**
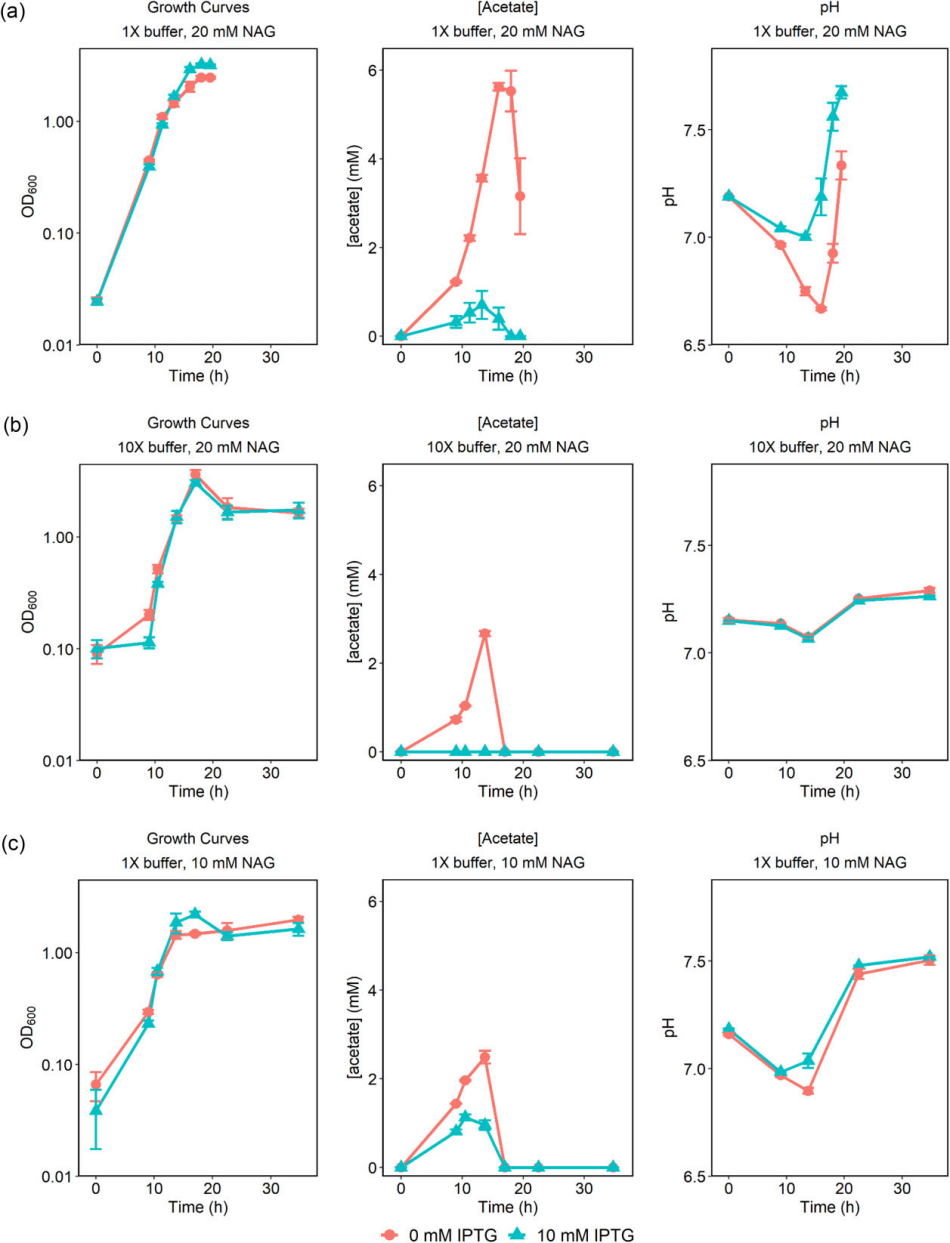
MR-1 cultures in 50 ml M5 minimal medium with and without 10 mM IPTG in 20 mM NAG growth curves (a), acetate concentrations (b), and pH (c). MR-1 cultures in 50 ml M5 minimal medium with and without 10 mM IPTG in 20 mM NAG + 10X buffer growth curves (d), acetate concentrations (e), and pH (f). MR-1 cultures in 50 ml M5 minimal medium with and without 10 mM IPTG in 10 mM NAG growth curves (g), acetate concentrations (h), and pH (i). Lines represent the average of three biological replicates and error bars represent standard error. *Y*-axes of growth curves are on a logarithmic scale.

We next investigated whether IPTG influenced metabolic product formation in *S. oneidensis* grown on NAG. *Shewanella oneidensis* was cultivated in 50 ml of minimal medium in 250-ml flasks with 20 mM NAG either with or without 10 mM IPTG (Fig. 2a). We observed a similar increase in carrying capacity in the presence of IPTG, although the effect was diminished in flasks versus in 96-well plates. We confirmed by microscopy that cell size was the same in both conditions, indicating that the difference in OD_600_ reflects a difference in cell density (Fig. S2). Samples from these cultures were analyzed via high performance liquid chromatography (HPLC) to detect changes in metabolic products. In cultures with IPTG, acetate accumulation was minimal and was quickly consumed (Fig. 2a). Without IPTG, approximately 5.75 mM of acetate accumulated before being consumed. NAG and IPTG co-eluted in our HPLC method and their peaks could not be resolved. However, no NAG/IPTG peak was detected after 18 hr in the no IPTG condition, and the peak area was equal to that of 10 mM IPTG in the IPTG-containing cultures after 16 hr. Further, we did not detect IPTG degradation as measured by galactose levels in the media. This indicates complete utilization of NAG both with and without IPTG. The pH trends of these cultures were consistent with the observed acetate concentrations, with the pH dropping when acetate concentrations were high (Fig. 2a). We also investigated the effect of IPTG on cultures grown with 40 mM d, l-lactate and found that IPTG had no effect (Fig. S3).

The acidification of the cultures and accumulation of acetate could be prevented when *S. oneidensis* was cultivated in the same conditions except with a 10X buffering capacity (Fig. 2b) or with half the original concentration of NAG (Fig. 2c). The difference in carrying capacity between +/− IPTG conditions was reduced with higher buffering capacity or lower starting NAG concentrations (Table S2), although acetate accumulation remained lower in the cultures with IPTG. This observation indicates that IPTG still significantly alters metabolism of *S. oneidensis* even when the growth appears unaffected. NAG was also completely utilized in cultures with either 10 mM NAG or with 10 X buffering capacity, and no IPTG degradation was detected. Taken together, the trends of growth, acetate accumulation, and culture pH suggest that acetate accumulation stalls growth of *S. oneidensis* and that IPTG counteracts the negative effects of acetate accumulation in the cultures.

**Fig. 3.**
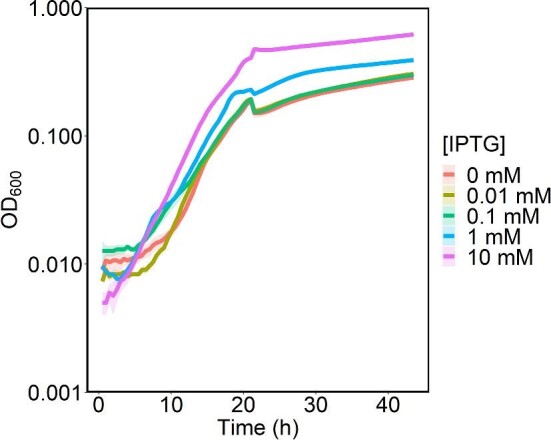
MR-1 growth curves in 200 µl M5 minimal medium with 20 mM NAG and varying IPTG concentrations. *Y*-axis is on a logarithmic scale. Lines represent the average of three biological replicates and transparent ribbons represent standard error.

IPTG at concentrations of 1.0 and 10 mM significantly increased the carrying capacity (*p* ≤ 0.05) for cultures with 20 mM NAG in 200 µl cultures in a 96-well plate (Fig. 3). However, IPTG concentrations of 1 mM or less did not enhance MR-1 growth on 20 mM NAG in 50-ml cultures (Fig. S4).

**Fig. 4.**
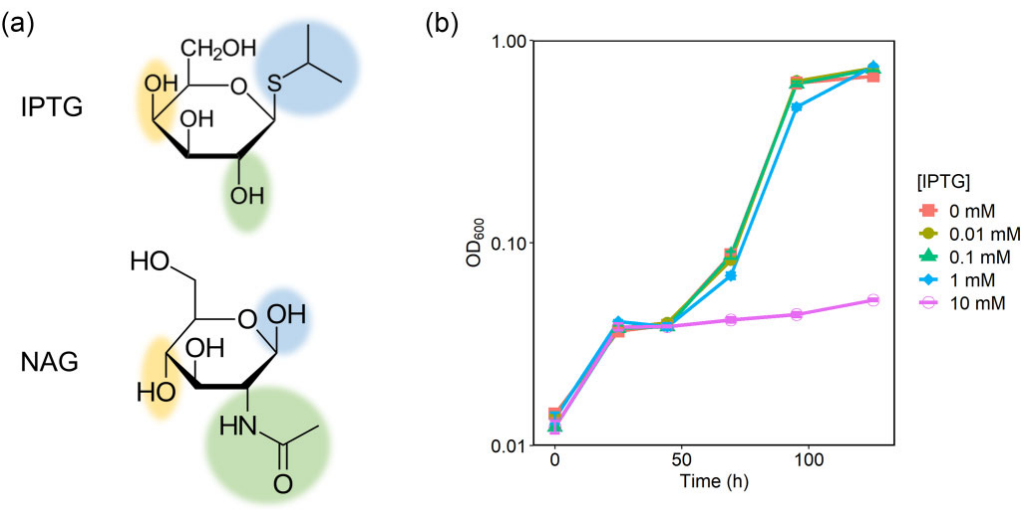
(a) The chemical structures of IPTG and NAG. Their structure differs in the highlighted regions. (b) Growth curves of *S. oneidensis* growth on 20 mM glucose with varying concentrations of IPTG. *Y*-axis is on a logarithmic scale. Lines represent the average of three biological replicates and error bars represent standard error.

Since IPTG is similar in structure to NAG (Fig. 4a), we hypothesized that IPTG induced expression of NAG metabolic genes. The key genes involved in metabolism of NAG are controlled by the NagR repressor, which is released from the operator site in the presence of NAG (Novichkov et al., 2013). Deletion of *nagR* in *S. oneidensis* results in a strain that is capable of growth on glucose due to the unregulated, constitutive expression of NAG permease and NAG kinase, which can perform the functions of the missing glucose permease and kinase to some degree (Chubiz & Marx, 2017). To determine whether IPTG induced the NagR regulon, we cultivated *S. oneidensis* in minimal media with 20 mM glucose and varying concentrations of IPTG. Our results show that higher IPTG concentrations diminish the ability for *S. oneidensis* to grow on glucose, suggesting that IPTG did not induce the NagR regulon (Fig. 4b). *Shewanella oneidensis* cannot natively grow on glucose; however, the ability to utilize glucose evolves quickly because only a single mutation is required for the phenotype to arise (Chubiz & Marx, 2017). This accounts for the growth observed on glucose after prolonged culturing.

## Discussion

Our results indicate that IPTG reduced acetate accumulation by MR-1 during growth on NAG. Rapid accumulation of acetate in the absence of IPTG was accompanied by a drop in culture pH. Acetate accumulation in *E. coli* cultures inhibits growth by causing decreased expression of TCA cycle and glyoxylate shunt genes (Orr et al., 2019). Acetate accumulation also appears to impact growth of *S. oneidensis* in our experiments, although it is not known whether acetate decreases expression of TCA cycle genes in this organism. Higher buffering capacity and lower NAG concentrations both reduced acetate accumulation to a rate that did not inhibit growth. With more buffering capacity, the pH presumably did not inhibit the expression of TCA cycle or glyoxylate shunt genes to the same extent as in cultures with the standard buffering capacity, thus allowing acetate to be more quickly metabolized. Lower NAG concentrations similarly prevent such a notable drop in pH; however, in this case it is due to less accumulation of acetate. The presence of IPTG in both cases displayed a compounding effect in which the acetate accumulation was even lower. These data point to a role of IPTG in improving acetate metabolism in *S. oneidensis* MR-1, perhaps through the induction of TCA cycle and/or glyoxylate shunt genes. Although we initially hypothesized that IPTG may interact with the native NagR regulator, another possibility is that it induces the HexR regulon, which includes genes of the glyoxylate shunt. Since the glyoxylate shunt is responsible for assimilating acetate as a carbon and energy source, its upregulation could increase the rate of acetate assimilation (Feng et al., 2012). Although outside the scope of this study, future work could explore the influence of IPTG on the HexR regulon and interaction with the HexR protein.

High-throughput experimentation with induction of heterologous genes in a 96-well plate is a common practice. Using this culturing method, we found that as little as 1 mM IPTG can cause a significant effect on growth. Higher IPTG concentrations of 10 mM were required for the effect to be seen in 250-ml shake flasks. While this is a high concentration of the inducer, it remains within the standard working range. The difference in required IPTG to observe the enhanced growth between these two culturing methods may be caused by oxygen availability. Larger cultures with more shaking are more efficiently aerated (Somerville & Proctor, 2013), and in *S. oneidensis* a higher availability of oxygen leads to a decrease in acetate production and higher carbon flux toward the TCA cycle (Tang et al., 2007). The differences in *S. oneidensis* metabolism caused by oxygen levels suggest that in the smaller cultures, acetate accumulates to an even greater level than we report from the shake flasks, potentially explaining the greater impact that IPTG has on growth in these conditions.

Unexpectedly, we found that at the 200 µl scale, *S. oneidensis* grown on 20 mM NAG or on 20 mM lactate without IPTG have similar final cell density, despite the greater size of the NAG molecule. We speculate that this was due to incomplete NAG utilization under this condition, although we could not measure NAG because of the small culture size. As culture size decreases, aeration and oxygen availability also decrease (Somerville & Proctor, 2013). Oxygen limitation reduces the substrate utilization of *S. oneidensis* (Pinchuk et al., 2010). At the 50 ml scale, we observe the expected growth yield difference between *S. oneidensis* growth on NAG vs d, l-lactate (Fig. S3).

To our knowledge, the phenotype of wild-type *S. oneidensis* displaying enhanced growth on NAG with IPTG has not been reported in the scientific literature. The enhanced growth of wild-type *S. oneidensis* on NAG when cultivated with IPTG is an important observation to be mindful of when testing inducible systems in this organism because of this inducer's capability of altering *S. oneidensis* metabolism. The impact of this was showcased by our discovery of the phenotype during experimentation with *S. oneidensis* CRISPRi control strains. This observation may also be buried in the literature and could raise questions regarding results without thorough controls. For example, Jeon *et al.* report optimal *n*-butanol production by an engineered strain of *S. oneidensis* on 2% NAG, 0.3% butyrate, and 0.1 mM IPTG under microaerobic conditions (Jeon et al., 2018). This strain overexpresses recombinant proteins CoA transferase, acetyl-CoA synthase, and alcohol dehydrogenase from an IPTG-inducible promoter. Since the carbon source was NAG, it is unclear whether the IPTG aided in *n*-butanol production because of expression of these proteins, or if it was decreasing acetate accumulation in the media. The *n*-butanol-producing strain generated the highest titers under microaerobic conditions. Our data suggests less aeration (therefore lower oxygen concentrations) exacerbates the effect of IPTG on NAG-cultivated *S. oneidensis*. So, one could speculate that this effect is responsible for the higher production of *n*-butanol under microaerobic conditions. We encourage researchers working with *S. oneidensis* to be mindful of these observations during their efforts to engineer this organism so that false positive correlations can be avoided.

## Materials and Methods

### Bacterial Strains and Culturing Conditions

Wild-type *Shewanella oneidensis* MR-1 was used for all experiments in this study. Plasmid pJMP2846 was used as the CRISPRi non-targeting control (Peters et al., 2019). *Shewanella oneidensis* was grown at 30°C with shaking in M5 minimal medium (1.29 mM K_2_HPO_4_, 1.65 mM KH_2_PO_4_, 7.87 mM NaCl, 1.70 mM NH_4_SO_4_,475 μM MgSO_4_·7H_2_O, 10 mM HEPES, 0.01% (w/v) casamino acids, 1 × Wolfe's mineral solution, and 1 × Wolfe's vitamin solution without riboflavin, pH 7.2). For cultures with 10X buffering capacity, HEPES concentration was increased to 100 mM. M5 was supplemented with either sodium d, l-lactate, NAG, or sodium acetate to final concentrations of 20 mM unless otherwise stated. IPTG was added to cultures at the specified concentrations. Growth experiments were conducted in either 96-well plates containing 200 µl of media or 250-ml shake flasks containing 50 ml of media. Culture volumes are specified for each experiment.

### OD_600_ and pH Measurement

OD_600_ was measured every 15–30 min using an H1M BioTek Plate Reader for 200 µl cultures. OD_600_ of 50-ml cultures were measured on an Eppendorf BioSpectrometer using a 1 ml culture sample in a disposable cuvette with a 1-cm pathlength (diluted 1:10 when OD_600_ was above 1.0).

A 2-ml culture sample was taken from 50-ml cultures for pH measurement. The sample was centrifuged at 8 000 rpm for 5 min to remove cells from the media. The pH of the supernatant was measured using a Mettler Toledo FiveEasy Plus FP20 pH meter.

### HPLC Analysis

HPLC samples were prepared by centrifuging 1 ml aliquots from 50 ml cultures at 16 100 rpm for 10 min in a Minispin Plus Eppendorf microcentrifuge. A total of 700 µl of the supernatant was transferred to a 2 ml glass HPLC vial. Sodium acetate standards were prepared at concentrations of 30, 20, 15, 7, and 3 mM in Milli-Q water. Samples were refrigerated at 10°C in the HPLC autosampler during the run.

HPLC analysis was performed on a Shimadzu 20A HPLC using an Aminex HPX-87H column (BioRad, Hercules, CA) with a Microguard Cation H^+^ guard column (BioRad, Hercules, CA) at 50°C. Analysis was conducted at a 0.6 ml min^−1^ flow rate in 5 mM sulfuric acid and a 30-min run time. HPLC eluent was prepared by diluting 98% HPLC-grade sulfuric acid solution in Milli-Q water. The eluent was degassed at 37°C for 3–5 days prior to use. Acetate was detected by refractive index (RID-20A). NAG concentration could not be measured due to co-elution with IPTG.

### Data Analysis

Growth and HPLC data were analyzed using RStudio using packages ggplot2, dplyr, grid, and gridExtra (Wickham, 2016;R Core Team, 2021; Wickham et al., 2022). Carrying capacity and growth rates of *S. oneidensis* cultures were determined using the R package “growthCurver” using default values (Sprouffske & Wagner, 2016).

### Microscopy

Samples (2 µl) of 50 ml growth curves were periodically imaged on a Leica DM 1000 LED microscope and images were taken from a Leica ICC50 W camera and Leica app. Average cell areas were calculated with Image J (Schneider et al., 2012).

## Supplementary Material

kuad018_Supplemental_File
