# Supplementary material for: A common inducer molecule enhances sugar utilization by Shewanella oneidensis MR-1
Source: J Ind Microbiol Biotechnol. 2023 Aug 3;50(1):kuad018. doi: 10.1093/jimb/kuad018 (PMC10549210; doi:10.1093/jimb/kuad018)
Supplement: kuad018_Supplemental_File [file kuad018_supplemental_file.pdf]

Supplementary Information

A common inducer molecule enhances sugar utilization by *Shewanella oneidensis* MR-1

Megan Gruenberg[1] and Michaela TerAvest[1]

[1]Department of Biochemistry and Molecular Biology, Michigan State University, East Lansing, MI, USA

Table S1. Growth rate and carrying capacity of *S. oneidensis* growth in 200 µl M5 minimal medium on 20 mM of different carbon substrates with or without 10 mM IPTG. Values were calculated from the growth curves using the R package "growthCurver" using default values.

**Substrate**	**Growth Rate (h^{-1})**		**Carrying capacity (OD_{600})**	
	- IPTG	+ IPTG	- IPTG	+ IPTG
NAG	0.181 ± 0.005	0.168 ± 0.005	0.372 ± 0.002	0.648 ± 0.004
Lactate	0.250 ± 0.01	0.266 ± 0.028	0.447 ± 0.003	0.322 ± 0.005
Acetate	0.120 ± 0.008	0.193 ± 0.017	0.175 ± 0.005	0.165 ± 0.004

Table S2. Growth rate and carrying capacity of *S. oneidensis* growth in 50 mL M5 minimal medium. Values were calculated from the growth curves using the R package "growthCurver" using default values.

**Conditions**	**Growth Rate (h^{-1})**		**Carrying Capacity (OD_{600})**	
	-IPTG	+IPTG	-IPTG	+IPTG
20 mM NAG<br>1X buffering capacity<br>**(Figure 2a)**	0.393 ± 0.062	0.560 ± 0.045	2.644 ± 0.162	3.345 ± 0.082
20 mM NAG<br>10X buffering capacity<br>**(Figure 2d)**	0.956 ± 0.663	1.034 ± 0.553	2.257 ± 0.255	2.072 ± 0.183
20 mM NAG<br>1X buffering capacity<br>**(Figure 2g)**	1.658 ± 0.085	1.759 ± 0.11	0.633 ± 0.163	1.326 ± 0.978

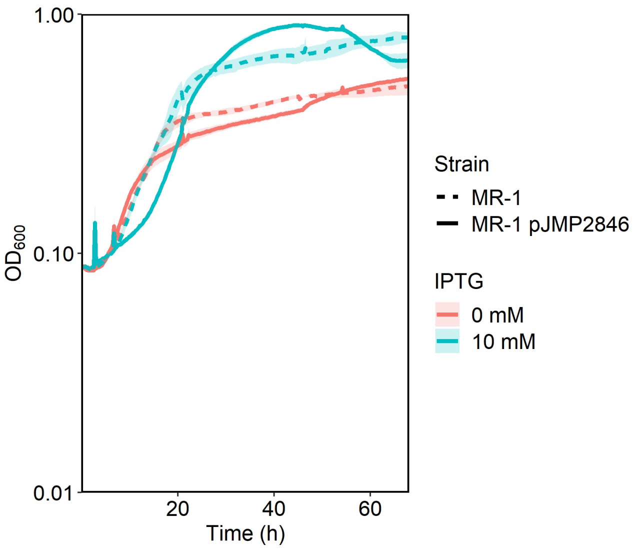

Figure S1. Growth of wild-type *S. oneidensis* and *S. oneidensis* CRISPRi non-targeting strain (MR-1 pJMP2846) in 200 µl minimal media with 20 mM NAG with or without 10 mM IPTG. Y-axis is on a logarithmic scale. Lines represent the average of three biological replicates and transparent ribbons represent standard error.

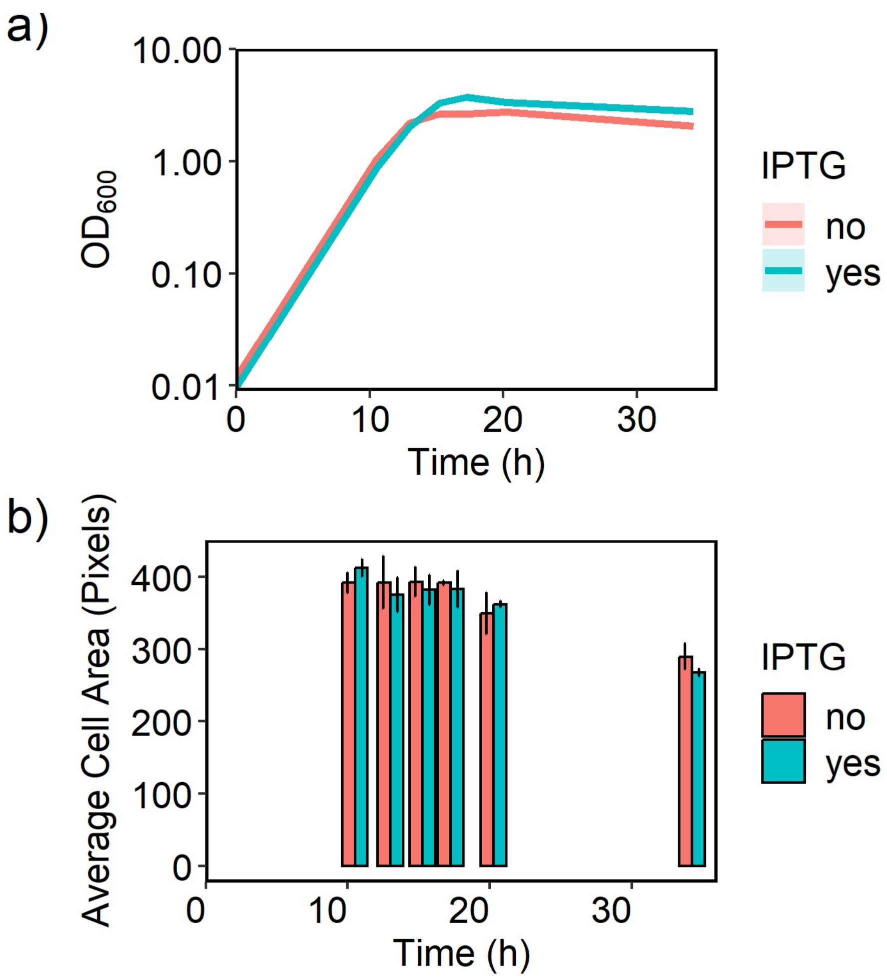

Figure S2. a) Growth curve of *S. oneidensis* MR-1 in 20 mM NAG with or without 10 mM IPTG in 50 ml cultures. b) CFU/ml of *S. oneidensis* MR-1 at 20.25 hours. c) Average cell area of *S. oneidensis* MR-1 at various times during the growth curve. +/- IPTG values at each timepoint are not significantly different (t-test, $p>0.05$).

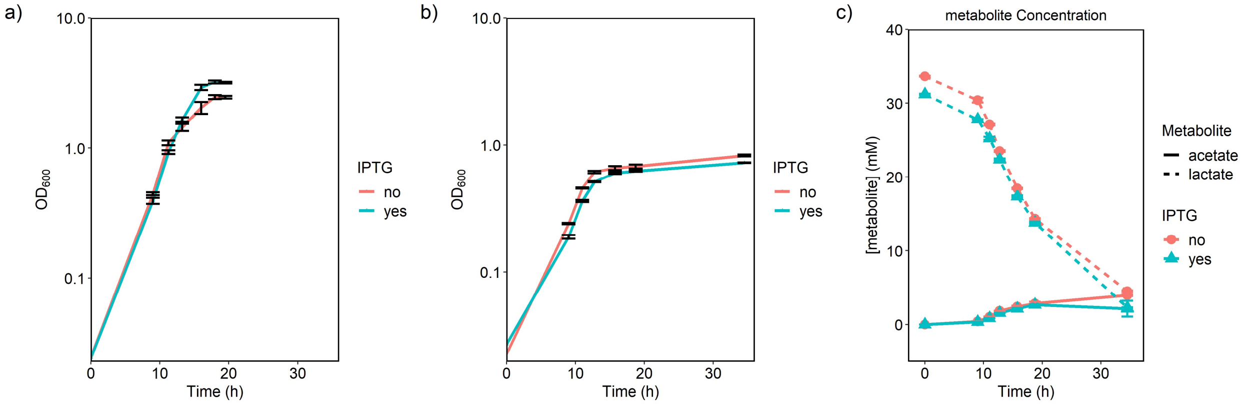

Figure S3. a) 50 ml growth curves of *S. oneidensis* MR-1 on 20 mM NAG with or without 10 mM IPTG. b) 50 ml growth curves of *S. oneidensis* MR-1 on 40 mM D,L-lactate with or without 10 mM IPTG. c) HPLC analysis of 50 ml *S. oneidensis* MR-1 cultures growing on 40 mM lactate with or without 10 mM IPTG.

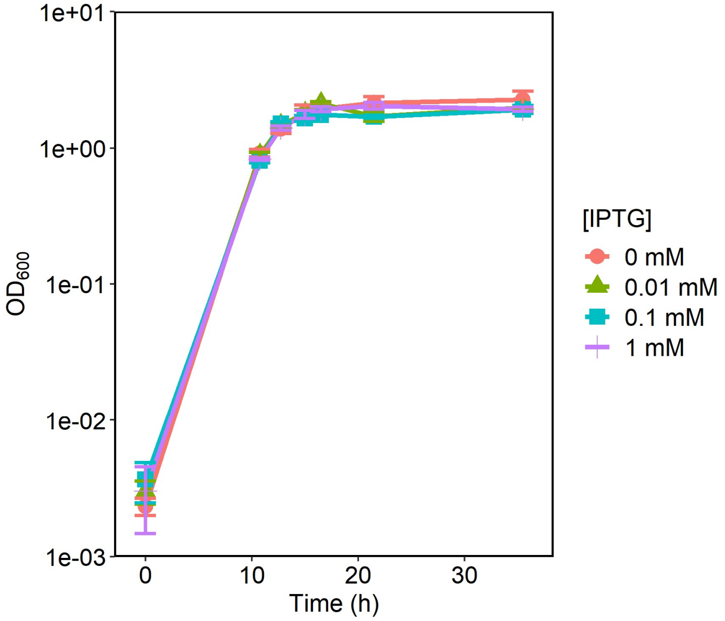

Figure S4. *S. oneidensis* growth curves in 50 ml M5 minimal medium with 20 mM NAG and varying IPTG concentrations. Y-axis is on a logarithmic scale. Lines represent the average of three biological replicates and error bars represent standard error.
